# The role of the NY-ESO-1 in the prognosis of gastric cancer

**DOI:** 10.17305/bb.2023.9937

**Published:** 2024-08-01

**Authors:** Zvonimir Misir, Goran Glavčić, Suzana Janković, Ivan Kruljac, Jakša Čugura-Filipović, Kristina Čimić, Monika Ulamec

**Affiliations:** 1 Department of Surgery, University Hospital Center “Sestre milosrdnice”, Zagreb, Croatia; 2 Department of Clinical Research and Biostatistics, Poliklinika Solmed d.o.o., Zagreb, Croatia; 3 Department of Gynecology, General Hospital “Dr Ivo Pedišić”, Sisak, Croatia; 4 Clinical Department of Pathology and Cytology “Ljudevit Jurak”, University Hospital Center “Sestre milosrdnice”, Zagreb, Croatia; 5 Department of Pathology and Center of Excellence for Reproductive and Regenerative Medicine, School of Medicine, University of Zagreb, Zagreb, Croatia

**Keywords:** Gastric cancer (GC), survival, New York esophageal squamous cell carcinoma-1 (NY-ESO-1), metastases

## Abstract

Gastric cancer (GC) is one of the most common malignancies worldwide and the fourth leading cause of cancer-related deaths. GC is a multifactorial disease influenced by both environmental and genetic factors. Its most critical features include invasiveness and high metastatic potential. Metastasis is a complex process, and our understanding of the mechanisms involved remains incomplete. Growing evidence suggests that cancer-testis antigens (CTAs) play a crucial role in the metastatic potential of various tumors. Several studies have linked CTA expression with lower tumor differentiation, higher metastatic potential, and poor chemotherapy response. New York esophageal squamous cell carcinoma-1 (NY-ESO-1) antigen, part of the CTA group, is expressed in tumor tissues, while its expression in normal tissues is restricted to spermatogonia. This study aimed to determine the expression of NY-ESO-1 in primary adenocarcinoma of the stomach, both with and without metastasis in regional lymph nodes, and to compare it with TNM stage, age, gender, and survival. We analyzed GC tissue from 53 node-negative and 55 node-positive primary gastric carcinoma patients for NY-ESO-1 expression using immunohistochemical assay. The results were correlated with clinicopathological parameters and survival. Patients with positive NY-ESO-1 expression in primary tumors had a median survival of 19.0 months (range 14.1–24.0), in contrast to those with negative expression, who had a median survival of 52.0 months (range 0.0–133.3) (chi-square 7.99, *P* ═ 0.005). T status, N status, and NY-ESO-1 expression were all independently associated with shorter survival. No significant difference in NY-ESO-1 expression in primary tumors was observed concerning lymph node metastasis status. In summary, our findings suggest that increased expression of NY-ESO-1 could potentially serve as a prognostic biomarker for GC.

## Introduction

A million new cases of gastric cancer (GC) are diagnosed annually, making it one of the most prevalent and lethal malignancies worldwide. According to the Global Cancer Observatory (GLOBOCAN) 2020 data, it rates fifth in incidence and fourth in cancer-related mortality worldwide [1, 2]. Although surgical resection remains the most effective treatment for locally aggressive GC, many patients present at an advanced stage [3]. They require intensive chemotherapy, which results in poor clinical outcomes [4], highlighting the need for novel prognostic and therapeutic methods.

Early invasion and metastasis are some of the defining characteristics of GC and entail a sequence of events known as the “invasion-metastasis cascade” [5]. DNA methylation changes are important in moderating gene signature and are recognized as a critical oncogenic mechanism in GC [6–8]. These epigenetic alterations activate cancer-testis antigens (CTAs) and play an active role in both early and advanced stages of carcinogenesis [9, 10]. Additionally, the tumor microenvironment (TME) represents a highly dynamic part of the tumor that is implicated in tumorigenesis. As an essential element of the TME, the tumor stroma affects tumor biology and contributes to cancer initiation, progression, metastasis, and therapeutic resistance [11].

CTAs are typically expressed in germ cells and placenta but not in mature normal tissue. In a proportion of solid human tumors, cancer-testis gene activation due to hypermethylation and, consequently, elevated protein expression have been identified via analysis of various types of human cancer [7, 12–14]. Some studies suggest that they can play a critical function in the progression of the cell cycle and in cell growth [15]. In addition, there is growing evidence that CTA expression correlates with tumors with a greater potential for malignancy, thus contributing to malignant behavior [13, 14, 16].

New York esophageal squamous cell carcinoma-1 (NY-ESO-1) belongs to the family of CTAs that is expressed in GC and is known to elicit an integrated humoral and cellular immune response in a high percentage of patients [13, 14]. The mRNA expression levels of NY-ESO-1 range from approximately 17% to 24% of GC patients, whereas, at the protein level, it was observed in up to 30% of GC patients using immunohistochemistry analysis [17–20]. NY-ESO-1 was initially identified in esophageal cancer and is highly immunogenic [12, 21]. Eventually, it was discovered that it was expressed in a variety of cancers, including neuroblastoma, malignant melanoma, hepatocellular carcinoma, breast cancer, and lung cancer [14, 16, 22].

It was demonstrated that approximately 75% of cancer patients express this antigen at some stage during their illness [13, 23, 24]. Due to its frequent expression in malignancies and ability to elicit integrated humoral and cellular immune responses, it is a promising immunotherapy target for cancer [12, 13, 25, 26]. In GC, a neutral effect of NY-ESO-1 expression on prognosis was demonstrated [17].

The purpose of this investigation was to evaluate the immunohistochemical expression of NY-ESO-1 in localized and regionally aggressive (lymph node-positive) primary GC specimens. Data were correlated to the relevant clinicopathological parameters and overall survival.

## Materials and methods

### Patients

From January 1, 2005 to November 30, 2015, tumor paraffin blocks from 108 patients with GC who did not receive neoadjuvant radio-chemotherapy were obtained from the Ljudevit Jurak Pathology Department Tumor Registry in Zagreb, Croatia. Samples of total gastrectomy with lymphadenectomy specimens were chosen. All patients with curable disease were treated by either D1-plus (T1N0 stage) or D2 lymphadenectomy with >15 lymph nodes harvested. There were 53 patients with N0 disease and 55 patients with N+ disease.

### Methods

Tumor tissue was fixed in 10% formaldehyde, and embedded in paraffin using routine procedures, from which 5 µm thin sections were cut and stained with hematoxylin and eosin (HE). All cases were routinely diagnosed by pathologists and met the WHO criteria for gastric adenocarcinoma, NOS. For each patient, a representative block of tumor tissue was chosen, and a tissue microarray (TMA) was conducted and analyzed on HE slides. Depending on lymph node status (negative/positive), tumors were divided into two categories.

The NY-ESO-1 primary monoclonal rabbit antibody was used (gift from Professor Spagnoli, University of Basel, Switzerland) [27, 28]. DAKO EnVisionTM+System, HRP (DAB) was used to visualize positive reactions according to the manufacturer’s instructions. Hematoxylin was used to counterstain the microscope specimens The immunohistochemical staining of the entire section containing at least 1000 tumor cells (TCs) was evaluated.

Two pathologists performed morphometric analysis for protein expression of investigated genes. A joint committee was responsible for settling all disagreements. The expression of proteins was analyzed in both epithelial and stromal cell compartments. Staining percentage was scored on a scale from 0 to 3; 0 (negative TCs); 1 (up to 10% positive TC); 2 (from 10% to 50% positive TC); and 3 (more than 50% positive TC). The intensity of staining was rated as 0-negative; 1-low, 2-medium, and 3-high. Semi-quantification of protein expression was represented by immunoreactivity score (IRS) which was calculated by multiplying staining percentage (0–3) and staining intensity (0–3) to create a range of 0–9. IRS was labeled as: 0 = negative; 1–4 = low; 5–9 = high.

Clinical and histopathological data (age, gender, tumor size, tumor type, TNM, vascular invasion, perineural invasion, and median survival) were obtained from the patient data archive of the University Hospital Center Sestre milosrdnice and the Croatian Institute of Public Health, with all necessary ethical approvals.

### Ethical statement

Ethics committee approval (EP-7811/16-9) was obtained from the University Hospital Center Sestre milosrdnice.

### Statistical analysis

Patient characteristics were evaluated using descriptive statistics. When applicable, continuous variables were presented as medians with interquartile ranges and compared using the Mann–Whitney and Kruskal–Wallis tests. Categorical variables were analyzed using the chi-square test with Yates correction. Spearman correlation coefficients were utilized for univariate analysis of coherence between immunohistochemistry parameters. Using the Kaplan–Meier curves and Log-Rank tests, the variance in patient outcomes was analyzed. To ascertain which parameters were independently associated with survival, a multivariate Cox regression analysis was conducted. A backward stepwise conditional approach was used in multivariate analysis. Receiver operating characteristic (ROC) analysis was conducted to determine the predictive ability of each variable in predicting 2-year survival. Two-sided *P* < 0.05 was considered statistically significant. IBM SPSS Version 20.0 was utilized for the statistical analysis.

## Results

### Univariate analyses

Our study included materials from 108 patients with gastric adenocarcinoma, of which the final outcome was known for 104 of them. Overall, the median age was 68 years (IQR 59–75) and median survival was 31.0 (IQR 13.3–48.7) months. Men and women were equally distributed between the groups (54 men and 54 women). Patients with lymph node metastases numbered 55 (50.9%). The most prevalent histological form of tumor identified by Lauren was mixed (49, 45.4%), followed by intestinal (38, 35.2%) and diffuse (17, 15.8%). The diameter of the tumor ranged from 3.0 to 7.0 cm (mean 4.1 cm). Patients with lymph node positivity were younger, had larger tumors, and had higher perineural and vascular invasion rates. Higher N status and perineural invasion were associated with younger age. As anticipated, a higher T and N status were associated with perineural and perivascular invasion. There was no significant association between gender and the analyzed parameters (Table 1).

**Table 1:** Univariate analysis of the study population based on lymph node metastases

		**Lymph node metastases**		
		**Absent**	**Present**	***P* value**
Vascular invasion, *n* (%)	Absent	44 (83)	30 (54.5)	0.001
	Present	9 (17)	25 (45.5)	
Perineural invasion, *n* (%)	Absent	45 (84.9)	23 (41.8)	<0.001
	Present	8 (15.1)	32 (58.2)	
Sex, *n* (%)	Female	30 (56.6)	24 (43.6)	0.178
	Male	23 (43.4)	31 (56.4)	
Tumor type, *n* (%)	Intestinal	25 (47.2)	13 (23.6)	0.013
	Diffuse	9 (17)	8 (14.5)	
	Mixed	16 (30.2)	33 (60)	
	Other	3 (5.7)	1 (1.8)	
T status, *n* (%)	1	21 (39.6)	2 (3.6)	<0.001
	2	15 (28.3)	3 (5.5)	
	3	14 (26.4)	43 (78.2)	
	4	3 (5.7)	7 (12.7)	
Age (years), median (range)	71 (63-76)	64 (56-72)	0.009	
Tumor size (cm), median (range)	3.0 (2.0-6.0)	5.5 (4.0-8.0)	0.001	
*NY-ESO-1 (epithelial), n (%)*				
0		15 (28.6)	14 (25.9)	0.787
1		2 (3.8)	1 (1.9)	
2		36 (67.9)	39 (72.2)	
*NY-ESO-1 (stromal), n (%)*				
0		17 (32.1)	16 (29.6)	0.784
1		36 (67.9)	38 (70.4)	

NY-ESO-1: New York esophageal squamous cell carcinoma-1.

NY-ESO-1 antigen was expressed by 75% (/108) of tested samples as measured by IHC (Table S1). There was no correlation of the NY-ESO-1 expression with the investigated clinicopathological parameters except T stage. Primary tumors with and without lymph node metastases showed no significant difference in NY-ESO-1 expression (*P* ═ 0.787, Table 1). The frequency of NY-ESO-1 antigen expression increased from the early stages (I and II, 1/30, 3.3%) to the advanced stages (IIIx002Band IV, 11/69, 15.9%), but the difference was not statistically significant (Table 1).

**Figure 1.**
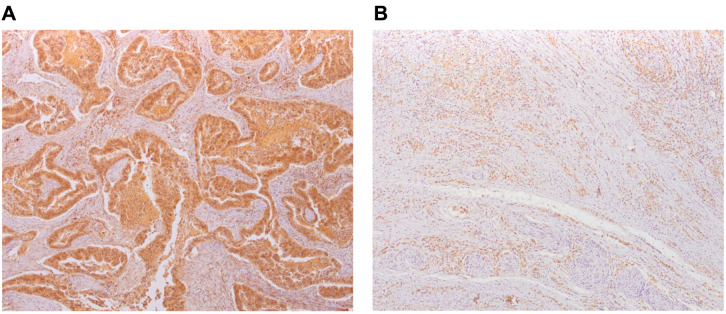
**Immunohistochemical staining.** (A) Strong epithelial NY-ESO-1 expression with weak stromal expression; (B) Strong epithelial NY-ESO-1 expression without stromal expression. NY-ESO-1: New York esophageal squamous cell carcinoma-1.

The expression of NY-ESO-1 in the carcinoma showed a strong association with the expression of NY-ESO-1 in the tumor stroma (*P* < 0.001). Since only two subjects had a moderate expression of NY-ESO-1 in epithelial cancer cells, we used the expression in the stroma in all subsequent multivariate analyses for a more streamlined data presentation and more accurate analyses (Table 2, Figure 1, and Table S1).

**Table 2:** Correlation of NY-ESO-1 expression in carcinoma and in stroma

		**Epithelial NY-ESO-1 expression**			***P* value**
		**Negative**	**Weak**	**High**	
**Stromal NY-ESO-1 expression, *n* (%)**	Negative	29 (100.0)	2 (66.7)	2 (2.7)	<0.001
	Weak	0 (0.0)	1 (33.3)	73 (97.3)	
	High	0 (0.0)	0 (0.0)	0 (0.0)	

NY-ESO-1: New York esophageal squamous cell carcinoma-1.

**Figure 2.**
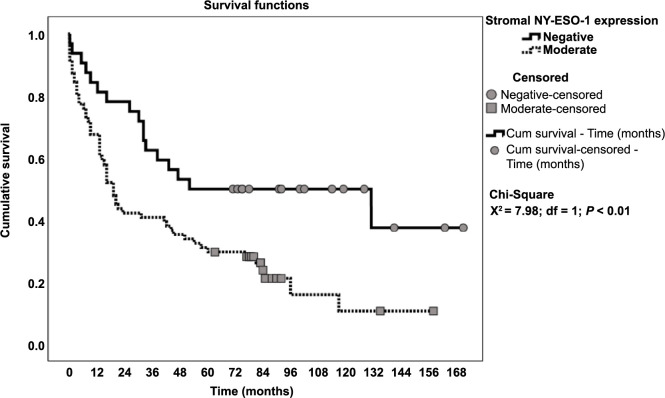
**Overall survival in all patients in relation to NY-ESO-1 expression.** NY-ESO-1: New York esophageal squamous cell carcinoma-1.

**Figure 3.**
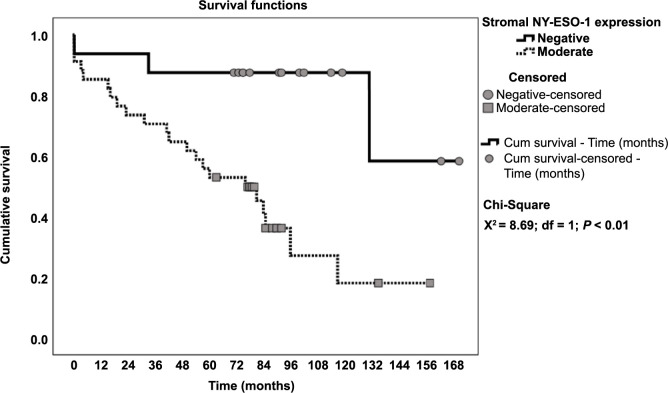
**Overall survival in patients without nodal metastases in relation to NY-ESO-1 expression.** NY-ESO-1: New York esophageal squamous cell carcinoma-1.

Patients with a weak expression of NY-ESO-1 in the stroma had a median survival of 19.0 (14.1–24.0) months, whereas patients with a negative expression had a median survival of 52.0 (0.0–133.3) months (chi-square 7.99, *P* ═ 0.005, Figure 2). The analysis of the association between NY-ESO-1 expression and survival in patients with and without lymph node metastases revealed that patients without lymph node metastases had a greater survival disparity (Figure 3 and Table S1).

### Multivariate models

Variables that showed statistical significance in univariate analysis were used in multivariate analysis. Individual variables were gradually introduced to the analysis, and the final step of the stepwise regression is shown in Table 2. For example, model 1 included only three variables (NY-ESO-1, age, and gender), of which only two were independently associated with survival. Tx002Band N status variables were added to model 2, and perineural and vascular invasion variables were added to model 3. As seen in the table, the expression of NY-ESO-1 in the stroma is associated with worse survival, independent of T and N status, age, or perineural invasion. All models are presented in Table 3.

**Table 3:** Multivariate analysis models

	**B**	**SE**	**Wald**	**HR**	**95% CI**		***P* value**
*Model 1 (age, sex, NY-ESO-1)*					
NY-ESO-1 expression (stromal)	0.806	0.281	8.241	2.238	1.291	3.879	0.004
Age	0.025	0.012	4.639	0.975	0.953	0.998	0.031
*Model 2 (age, sex, NY-ESO-1, T and N status)*					
NY-ESO-1 expression (stromal)	0.590	0.290	4.135	1.804	1.022	3.187	0.042
T status	0.359	0.169	4.499	1.432	1.028	1.995	0.034
N status	0.484	0.118	16.700	1.623	1.287	2.047	0.000
*Model 3 (age, sex, NY-ESO-1, T and N status, perineural and vascular invasion)*					
NY-ESO-1 expression (stromal)	0.693	0.283	5.987	2.000	1.148	3.485	0.014
N status	0.528	0.115	21.152	1.696	1.354	2.125	0.000
Perineural invasion	0.706	0.276	6.549	2.025	1.180	3.476	0.010

HR: Hazard ratio; CI: Confidence interval; NY-ESO-1: New York esophageal squamous cell carcinoma-1.

### Predicting 2-year survival

**Figure 4.**
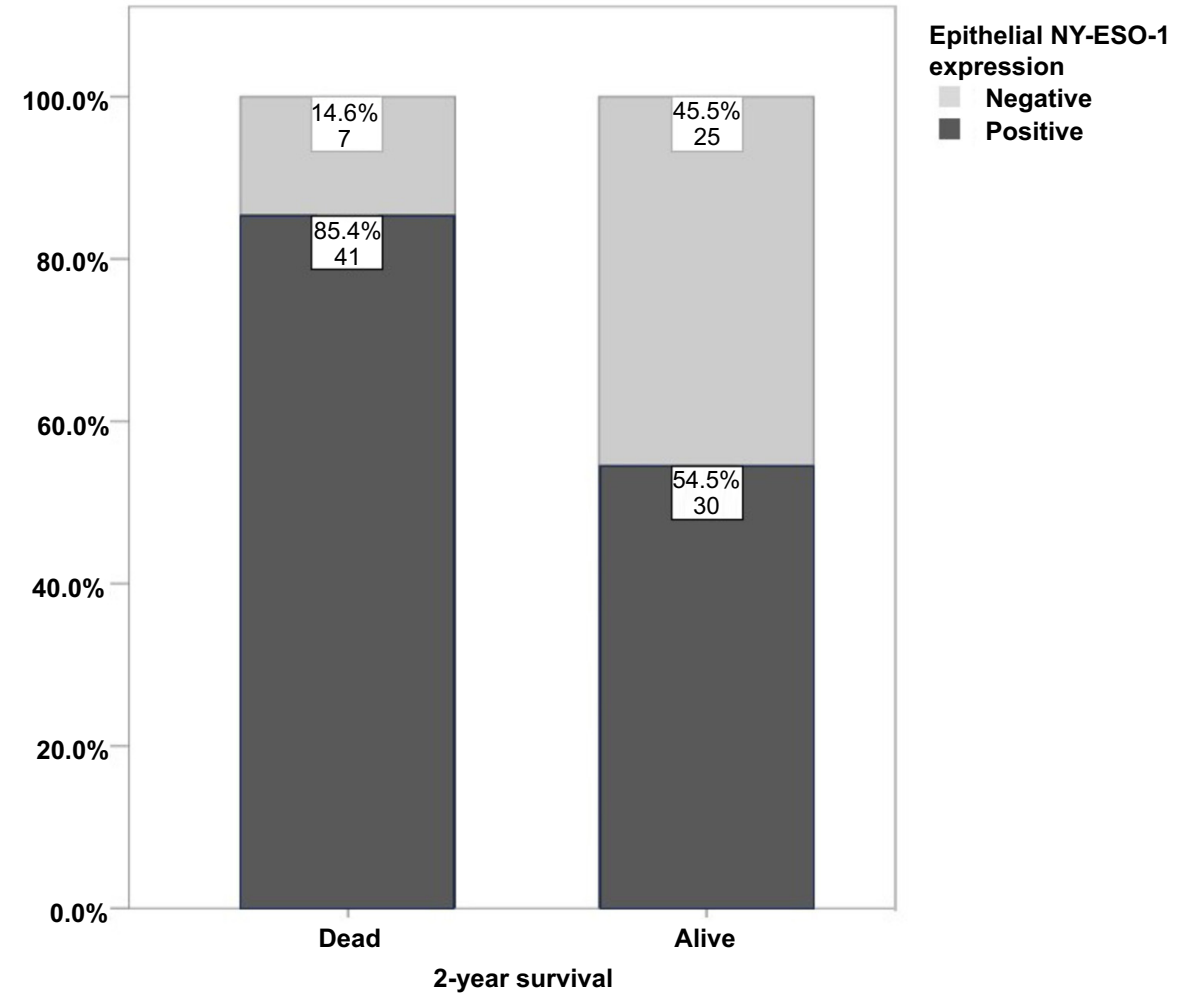
**Two-year survival rate in all patients correlated to epithelial NY-ESO-1 expression.** NY-ESO-1: New York esophageal squamous cell carcinoma-1.

Tumor size, stage, T status, and N status were all able to predict 2-year survival, but N status had the highest diagnostic accuracy (79.0%) of the four. Stromal NY-ESO-1 expression could predict 2-year survival with 65.4% accuracy. When including all variables associated with 2-year survival in the multivariate model, we concluded that only N status, tumor size, and expression of NY-ESO-1 in the stroma were independently associated with 2-year survival. Analyzing differences in 2-year survival, patients with high NY-ESO-1 expression were more likely to die (Figure 4).

## Discussion

NY-ESO-1 expression has been reported to correlate with advanced disease characteristics, such as aggressive cancer phenotype and clinical stage, across different tumor types [13, 15, 23–26]. Furthermore, it has been observed that NY-ESO-1 expression increases across disease stages, with a lower frequency in primary tumors than in metastases [13, 25, 27, 28]. Additionally, it was found that the magnitude of a NY-ESO-1-specific humoral immune response increases with disease progression and decreases with disease regression [29, 30].

The majority of studies analyzed the expression of NY-ESO-1 antigen via the presence of NY-ESO-1 mRNA (RT-PCR) [11, 19, 22]. Since posttranscriptional gene expression control can contribute to substantial discordance between mRNA and protein expression in cancer cells, the protein level (IHC) analysis is more clinically relevant (23). Other studies have reported mRNA expression data varying in protein expression levels as analyzed by immunohistochemistry [19, 22]. Some other factors differing among different studies included the use of different antibodies and antigen-retrieval techniques, as well as differences in patient selection. There are few studies reporting the expression of NY-ESO-1 at the protein level in patients with GC, as well as its impact on survival [22, 25, 31, 32]. It has been reported to be expressed in up to 20% of patients with metastatic GC having the capacity to induce both natural antibody and T cell responses [12]. Wang et al. [24] studied 101 GC specimens and found NY-ESO-1 mRNA expression level to be present in 11.9% of samples with an expression frequency that increased from 3.3% of stage I and II gastric tumors to 15.9% of stage III and IV tumors. In the same study, seven of the 12 NY-ESO-1 mRNA-positive samples were also positive for NY-ESO-1 protein. Fujiwara et al. detected NY-ESO-1 protein expression by IHC in 19/60 GC samples and increased NY-ESO-1 humoral response in advanced disease stages. Additionally, the authors conclude that NY-ESO-1 humoral immune response could be used as a marker for detecting advanced GC [22].

To our knowledge, this is the first study investigating the immunohistochemical expression of NY-ESO-1 in primary GC in relation to lymph node metastases and overall survival. We evaluated the frequency of NY-ESO-1 expression in cancer epithelial cells as well as stromal myofibroblasts, its clinicopathological significance, and its prognostic influence in primary GC. Our research revealed epithelial NY-ESO-1 positivity in 78 out of 108 (72%) cases of GC. This contrasts with previous investigations which reported a much lower expression of NY-ESO-1 in patients with GC [19, 22, 31]. Out of 78 positive GC specimens, 75 samples had high NY-ESO-1 epithelial expression which correlated with weak expression in tumor stroma. Different antibody clones used for NY-ESO-1 detection and the heterogeneous expression of NY-ESO-1 antigen in GC may contribute to the discrepancies between studies [13, 22, 33]. In our study, we observed a higher frequency of NY-ESO-1 expression in advanced disease stages. The data were not statistically significant, and there was no distinction between lymph node metastatic and nonmetastatic disease. However, it revealed an upward trend in NY-ESO-1 expression with increasing disease stage. In addition, we found an association between NY-ESO-1 expression and tumor invasion depth (T stage). As a prognostic biomarker for GC, NY-ESO-1 expression remains controversial. In some malignancies, its expression has been linked to poor clinical outcomes, but frequently, there was no correlation found [34, 35].

Fujiwara et al. analyzed NY-ESO-1 protein expression and humoral response in patients with GC and found that neither humoral response nor NY-ESO-1 expression had an impact on the overall survival rate. In the same study, however, the NY-ESO-1 humoral immune response in conjunction with carcinoembryonic antigen and CA19-9 tumor markers proved to be a useful tumor marker for detecting advanced GC [22]. In the present study, patients with a positive NY-ESO-1 expression in primary GC had a shorter overall survival, regardless of the status of lymph node metastasis. A more significant impact on survival was evident in patients without nodal metastases, suggesting these tumors’ increased malignant potential. In addition, NY-ESO-1 expression correlates between epithelial and stromal components and is an independent predictor of 2-year survival in patients with GC. Although the data on the prognostic significance of NY-ESO-1 expression are equivocal, we demonstrated the impact of NY-ESO-1 expression in early-stage disease on GC patients’ overall survival.

This investigation was limited by its retrospective nature, small sample size, and the fact that all cases originated from a single institution. Since CT expression in cancer is often heterogeneous, IHC-based analysis is likely to have a different detection rate due to sampling errors as compared to RT-PCR.

## Conclusion

Tumor NY-ESO-1 expression, and T and N status were all independently associated with survival in GC patients. NY-ESO-1 could predict 2-year survival in early-stage GC with 65.7% accuracy.

## Supplemental data

**Table S1.** Excel table showing immunohistochemistry results of NY-ESO-1 staining (intensity of staining, percentage of stained cells, IRS) in carcinoma cells and stromal myofibroblasts, as well as perineural and vascular invasion, size of the tumor, TNM data, stage, classification by Lauren, age, gender and survival data.

Available at the following link:

https://www.bjbms.org/ojs/index.php/bjbms/article/view/9937/3300

Staining percentage: 0 (no staining); 1 (up to 10% positive cells tumor/stroma [TC]); 2 (10% to 50% positive TC); 3 (more than 50% positive TC); The intensity of staining: 0 (negative); 1 (low), 2 (medium), and 3 (high). Immunoreactivity score (IRS) is calculated by multiplying staining percentage (0–3) and staining intensity (0–3). IRS was labeled as: 0 (negative); 1–4 (low); 5–9 (high). NY-ESO-1: NY-ESO-1: New York esophageal squamous cell carcinoma-1; IRS: Immunoreactivity score.
